# Identification of a Fusarium ear rot resistance gene in maize by QTL mapping and RNA sequencing

**DOI:** 10.3389/fpls.2022.954546

**Published:** 2022-09-13

**Authors:** Yusheng Xia, Baobao Wang, Lihong Zhu, Wenqi Wu, Suli Sun, Zhendong Zhu, Xinhai Li, Jianfeng Weng, Canxing Duan

**Affiliations:** ^1^National Key Facility for Crop Gene Resources and Genetic Improvement, Institute of Crop Sciences, Chinese Academy of Agricultural Sciences, Beijing, China; ^2^Shijiazhuang Academy of Agricultural and Forestry Sciences, Shijiazhuang, China

**Keywords:** maize, ear rot, *Fusarium verticillioides*, resistance gene, QTL mapping, RNA sequencing

## Abstract

Fusarium ear rot (FER) caused by *Fusarium verticillioides* is a prevalent maize disease. To comprehensively characterize the genetic basis of the natural variation in FER resistance, a recombinant inbred line (RIL) population was used to map quantitative trait loci (QTL) for FER resistance. A total of 17 QTL were identified by linkage mapping in eight environments. These QTL were located on six chromosomes and explained 3.88–15.62% of the total phenotypic variation. Moreover, *qFER1.03* had the strongest effect and accounted for 4.98–15.62% of the phenotypic variation according to analyses of multiple environments involving best linear unbiased predictions. The chromosome segment substitution lines (CSSLs) derived from a cross between Qi319 (donor parent) and Ye478 (recurrent parent) were used to verify the contribution of *qFER1.03* to FER resistance. The line CL171, which harbored an introgressed *qFER1.03*, was significantly resistant to FER. Further fine mapping of *qFER1.03* revealed that the resistance QTL was linked to insertion/deletion markers InDel 8 and InDel 2, with physical distances of 43.55 Mb and 43.76 Mb, respectively. Additionally, *qFER1.03* differed from the previous resistance QTL on chromosome 1. There were three annotated genes in this region. On the basis of the RNA-seq data, which revealed the genes differentially expressed between the FER-resistant Qi319 and susceptible Ye478, *GRMZM2G017792* (MPK3) was preliminarily identified as a candidate gene in the *qFER1.03* region. The Pr-CMV-VIGS system was used to decrease the *GRMZM2G017792* expression level in CL171 by 34–57%, which led to a significant decrease in FER resistance. Using RIL and CSSL populations combined with RNA-seq and Pr-CMV-VIGS, the candidate gene can be dissected effectively, which provided important gene resource for breeding FER-resistant varieties.

## Introduction

Maize (*Zea mays* L.) is widely grown from the Northeast Plain to the Yunnan–Guizhou Plateau in the southwestern part of China. It is an important food crop, feed crop, and a source of energy in China, making it a key contributor to agricultural production and the national economy. Ear rot caused by many pathogenic fungi is one of the most destructive diseases of maize, and often leads to a considerable decrease in yield and quality (Duan et al., 2016; Lanubile et al., 2017; Zhou et al., 2018). The previous researches identified the *Fusarium verticillioides* and *Fusarium graminearum* species complex as the dominant pathogens responsible for maize ear rot in China (Shi and Bai, 1992; Qin et al., 2014; Zhou et al., 2016; Xiao et al., 2017; Sun et al., 2017a,b; Du et al., 2019; Wei et al., 2019). Maize ear rot caused by *F. verticillioides* (FER) is widespread in temperate and semitropical areas, including Asian, American, and European maize-growing areas. *F. verticillioides* can produce dangerous fumonisins that are toxic for humans and animals (Munkvold, 2003; Duan et al., 2016). This fungus overwinters in soil, seeds, and plant residues and spreads to maize grains through the roots and the air (Morales-Rodríguez et al., 2007; Chen et al., 2016). Additionally, it typically grows as white–pink mycelia on the seeds and/or silk. Infected kernels may also exhibit “starburst” symptoms, in which white stripes radiate from the silk-attachment site at the cap or from the base of the kernel (Lanubile et al., 2017).

The most economical and effective method for controlling FER involves the breeding and cultivation of resistant varieties (Duan et al., 2015a). Introducing disease resistance-related quantitative trait loci (QTL) or genes from donors into superior maize germplasm is a feasible strategy for developing disease-resistant germplasm and commercial hybrids (Nankam and Pataky, 1996). Some studies have shown that maize resistance to FER is typically a quantitative trait (Duan et al., 2015b). Screening resistant resources and mapping resistance genes or QTL are the basis for resistance breeding. The considerable abundance of genetic material and the strong influence of environmental factors have delayed the accurate localization of QTL, which has had a detrimental effect on the efficiency of marker-assisted selection (MAS) during breeding (Robertson-Hoyt et al., 2006). Increasing the population size and the number of molecular markers, improving the accuracy of the phenotype-based identification of ear rot, and integrating data from multiple environments will help overcome such limitations. Identifying and mapping resistance genes or QTL that can be stably expressed in different environments is critical for developing disease-resistant varieties. Several of the studies on FER that have been conducted over the past 30 years identified effective QTL for FER resistance (Yang et al., 2010; Xiang et al., 2012). Studies on the genetic structures and variations associated with complex maize traits have usually involved linkage analyses. However, most of the available genetic maps were constructed on the basis of low-throughput and low-density molecular markers, which has limited the efficiency and accuracy of QTL mapping as well as the coverage of genetic markers (Holland, 2007). Compared with simple sequence repeat (SSR) markers used for mapping, next-generation sequencing technology for genotyping is a powerful tool for developing single nucleotide polymorphism (SNP) markers and constructing high-density genetic maps. Using recombinant inbred line (RIL) populations with ultra-high-density genetic maps is an effective method for identifying QTL for complex agronomic traits (Zhou et al., 2016; Zhang et al., 2017). Accordingly, they have been widely used for mapping stress resistance QTL/genes in maize (Ding et al., 2008; Wang et al., 2018). However, the genetic background of a RIL population can be complex and easily affected by environmental factors (Eshed and Zamir, 1994; Alonso-Blanco and Koornneef, 2000). Chromosome segment substitution lines (CSSLs) can minimize the interference from the genetic background, making them suitable for mapping, confirming, and cloning target genes and QTL. A CSSL can also improve the accuracy of QTL mapping by separating single or several chromosomal fragments from the donor parent with the same genetic background as the recurrent parent. Additionally, CSSLs have been used to detect QTL with relatively small additive effects that have been masked by QTL with larger additive effects in primary populations (Shim et al., 2010).

Combined with QTL mapping, transcriptome sequencing is a useful technique for identifying candidate genes and validating loci for quantitative traits (Korte and Farlow, 2013). Combining these methods has enabled researchers to overcome the limitations of either method performed alone (Brachi et al., 2010).

Virus-induced gene silencing (VIGS) exploits RNA-mediated antiviral defense mechanisms, namely RNA interference and post-transcriptional gene silencing, to downregulate gene expression, which often results in a “functional deficit” phenotype in plants (Vance and Vaucheret, 2001; Becker and Lange, 2010). Although several VIGS vectors have been developed for maize, their utility is limited because of several factors (e.g., inefficient viral infection, unstable insertion, relatively short gene-silencing period, inappropriate inoculation methods, and abnormal growth temperature requirements). A maize gene-silencing system was recently established on the basis of the cucumber mosaic virus (CMV) to overcome many of the limitations of existing VIGS systems developed to silence maize genes (Li et al., 2021).

Most previously reported QTLs and candidate genes exhibit a minor effect on FER resistance in maize. This indicates that pyramiding of QTLs or genes from different resources would be an effective approach to improve FER resistance. Therefore, exploration of more effective and stable QTLs that impart FER resistance is necessary to provide resources for molecular breeding of resistant varieties of maize. In the present study, the maize inbred lines Qi319 and Ye478 were selected as the resistant and susceptible parents, respectively, to generate a RIL population for QTL mapping. An ultra-high-density bin map with 4,183 SNP markers was constructed along with the RIL population (Zhou et al., 2016). The relevant CSSLs were developed for fine mapping and for evaluating FER resistance to confirm and refine mapped region. The aims of this study were to finely map FER resistance gene in Qi319 and obtain the candidate gene in the targeted QTL region. The results will provide new molecular markers and genetic resource to aid in the breeding of FER-resistant varieties.

## Materials and methods

### Plant materials and pathogen

A RIL population consisting of 300 F_11_ lines was developed by single-seed descent from a cross between maize inbred lines Qi319 and Ye478 (Zhou et al., 2016). Inbred line Qi319 was developed from the FER-resistant maize hybrid 78,599 by the Shandong Academy of Agricultural Sciences (Tian et al., 2014). In contrast, Ye478 is a FER-susceptible inbred line with excellent agronomic traits and high general combining ability. Additionally, CSSLs were constructed using Qi319 and Ye478 as the donor and receptor parents, respectively (Lu et al., 2020). After back-crossing for five generations and self-crossing for three generations, the following 12 CSSLs were selected to verify the FER resistance QTL on chromosome 1: CL160, CL171, CL9, CL172, CL173, CL82, CL14, CL174, CL45, CL61, CL28, and CL21 (i.e., Types 1–12). Type 2 (CL171) was the CSSL with the candidate FER resistance-related gene. The F_2_ hybrid generation and the F_3_ selfing generation derived from CL171 and Ye478 were used for the subsequent gene mapping, whereas CL171 was used for the VIGS-based silencing of the candidate FER resistance-related gene.

The FER pathogen *F. verticillioides* strain used for inoculation, FVHN-10, was isolated from Zhumadian, Henan Province, and then was purified and stored in the laboratory of the Institute of Crop Sciences, Chinese Academy of Agricultural Sciences (Duan et al., 2016). FVHN-10 was proved be strong virulence to susceptible maize ears, such as Ye478, B73, and Ye107.

### Phenotypic evaluation of maize for FER resistance

In May 2019, 300 RIL materials were grown in Changping (Beijing), Shunyi (Beijing), and Xinxiang (Henan). In May 2020, they were grown repeatedly in Changping (Beijing) and Xinxiang (Henan). In May 2021, they were grown in Shunyi and Changping (Beijing) and Xinxiang (Henan). Each material was grown in a row (5 m long), with 0.6 m separating rows and 0.25 m separating plants. Each row comprised 25 plants. As controls, Qi319 (resistant) and Ye478 (susceptible) were acted as controls for every 50 rows. To produce the inoculum, *F. verticillioides* was grown on potato dextrose agar (PDA) medium in plates, which were incubated in darkness. A substantial abundance of conidia was produced in about 10 days. Six days before plants were inoculated, the PDA medium covered with mycelium and conidia was cut into small pieces in an ultra-clean platform and transferred to sterilized potato dextrose broth (PDB) medium for the subsequent propagation. The filtered *F. verticillioides* conidial suspension was diluted to 1 × 10^6^ spores/mL. The silk channel injection method was used to inoculate maize ears at 5–7 days after silk emergence when silks are elongated, pollinated, and may have some tip browning but are not dry (Duan et al., 2022). Briefly, the continuous syringe was adjusted and then inserted along the side of the corn silk to inject 2 ml conidial suspension into each ear through the silk channel. At about 40 days after the inoculation, maize ears were hand husked and removed line by line. FER incidence and severity were surveyed and resistance scales were evaluated (Wang, 2005; Duan et al., 2022). In this study, the severity of FER was accurately determined on the basis of the diseased ear area, which was calculated using the Automatic Imaging System for Ear Rot (customized by the National Agricultural Information Engineering Technology Research Center). The average diseased ear area (%) of each line was used for further analyses.

### Analysis of phenotypic data and heritability

All descriptive statistics (mean, range, skewness, and kurtosis) of the parental lines and RILs across the eight environments were analyzed using the Statistical Product and Service Solutions (SPSS) software v20.0. The generalized heritability (H2) of FER resistance in the eight environments was analyzed using the method of Wang (2017). Standard analysis of variance with the general linear model procedure (PROC GLM) was adopted to estimate all variance in Excel. We used the following genetic model: *H^2^ = σ_G_^2^*/(*σ_G_^2^ + σ_G𝜀_^2^*/*e + σ_𝜀_^2^*/*er*), where σ_G_^2^ is the genetic variance reflecting the blocking effect, σ_𝜀_^2^ is the error variance, σ_G𝜀_^2^ is the genotype × environment interaction, and *e* and *r* are the number of environments and repeats, respectively (Knapp et al., 1985). Inclusive composite interval mapping (ICIM) was used to locate QTL (Wang, 2009).

### QTL mapping for FER resistance in the RIL population

The QTL for FER resistance were analyzed using the ICIM method in the QTL IciMapping software (version 4.0; Wang, 2009). The ICIM method can exclude the influence of QTL outside the current interval. Specifically, this method involves a two-step strategy to effectively separate the cofactor selection from the interval mapping process, thereby controlling the background additive and dominance effects more effectively than compound interval mapping and improving the identification of QTL with additive effects (Zeng, 1993). The best linear unbiased prediction (BLUP) values were calculated with the following model: BLUP = (μ1 + …… + μ8)/*e*, where μ1–μ8 are the average performances in E1 (2019 in Shunyi), E2 (2019 in Changping), E3 (2019 in Xinxiang), E4 (2020 in Xinxiang), E5 (2020 in Changping), E6 (2021 in Shunyi), E7 (2021 in Changping), and E8 (2021 Xinxiang), respectively, and *e* is the number of environments (Wang, 2017). For each dataset, the significance threshold for affirming a putative QTL was obtained from 1,000 permutations was as follows: *p* < 0.05 with a logarithm of odds (LOD) score > 3.5 (Doerge and Churchill, 1996).

### Validation of the FER resistance loci in CSSL populations

After generating a CSSL population with elite inbred line Qi319 as the donor and Ye478 as the receptor, we obtained 200 CSSLs covering all maize chromosomes, from which 12 CSSLs (CL160, CL171, CL9, CL172, CL173, CL82, CL14, CL174, CL45, CL61, CL28, and CL21) covering chromosome 1 were selected to identify *qFER1.03*.

To accurately locate the FER resistance-related gene on chromosome 1, insertion/deletion (InDel) markers were designed to increase the marker density between 40.0 and 50.0 Mb (B73 RefGen_v3). A PCR amplification was performed using the GeneAmp 9,700 PCR System (ABI, Norwalk, CT, United States). The 20-μL reaction volume consisted of 50 ng genomic DNA, 0.2 μm primer mix, 1.5 U Taq DNA polymerase, 0.2 mm dNTPs, and 2.0 μl 10 × buffer. The PCR program was as follows: 94°C for 4 min; 35 cycles of 94°C for 1 min, 50–60°C for 1 min, and 72°C for 1 min; 72°C for 10 min.

### RNA-seq analysis

Total RNA was isolated from the ears of Qi319 and Ye478 plants inoculated with *F. verticillioides* (12 and 72 h) and the uninoculated controls (0 h, CK) using the TRIzol Kit (Invitrogen, Carlsbad, CA, United States) and then purified using the RNeasy kit (Qiagen, Germany). Three replicates were prepared for each sample. The purified RNA was used to construct a cDNA library using the NEBNext Ultra RNA Library Prep kit. The 18 libraries were subsequently sequenced using the Illumina HiSeq 2,500 system. After removing adapter sequences and low-quality reads, the high-quality paired-end reads were mapped to the maize reference genome (B73_v3) using the spliced read mapper TopHat (version 2.0.12; Kim et al., 2013). A principal component analysis was performed using the default settings of the prcomp function in the R software to interpret the relatedness among all replicates for each genotype. Significant differentially expressed genes (DEGs) were determined on the basis of a 2-fold expression-level change and a false discovery rate < 0.05 using the Cuffdiff module of Cufflinks (Trapnell et al., 2012).

### Construction of target gene VIGS vectors

The Pr-CMV-VIGS system was used to silence the target gene (Li et al., 2021). Briefly, the target gene (150–200 bp) was amplified by RT-PCR using primers containing LIC adapters. The PCR product was purified and treated with T4 DNA polymerase (Thermo Scientific) in the presence of 5 mm dATP (Promega) at 37°C for 30 min before inactivating the T4 DNA polymerase at 75°C for 20 min. The pCMVZ2_2bN81_-LIC vector was digested with *Apa*I and then similarly treated with T4 DNA polymerase in the presence of 5 mm dTTP (Promega). The T4 DNA polymerase-treated PCR product and pCMVZ2_2bN81_-LIC vector were mixed and incubated at 70°C for 10 min and then at 22°C for 30 min. The mixture was subsequently used to transform *Escherichia coli* DH5α competent cells according to a heat shock method. The transformants were verified by sequencing, which was performed by the Genomic Sciences Laboratory at Sangon Biotech. The amplified product and two other CMV RNA sequences were inserted into *Agrobacterium tumefaciens* GV3101 cells, which were then grown overnight at 28°C in LB medium containing 50 mg/l kanamycin and 50 mg/l rifampicin. Equal amounts of GV3101 cells containing CMV RNA 1, 2, and 3 constructs were mixed, collected by centrifugation, and resuspended in infiltration buffer (10 mm MgCl_2_, 10 mm MES pH 5.6, and 0.2 mm As) for an optical density at 600 nm of 1.0. The cell solutions were left undisturbed at room temperature for 3 h. They were then injected into the unfolded leaves of 5-week-old *Nicotiana benthamiana* plants using a syringe. The plants were incubated in darkness for 48 h and then placed under light. At 7 days after the inoculation, *N. benthamiana* leaves were collected, juiced, and used to inoculate the heart leaves of maize seedlings at the two-leaf and one-heart stage by rubbing. The inoculated leaves were covered with plastic wrap and then the maize seedlings were incubated in darkness for 48 h. The plastic wrap was removed and seedlings were transferred to the field and cultivated normally (Li et al., 2021).

### Validation of the RNA silencing effect by quantitative real-time PCR (qRT-PCR)

Total RNA samples served as the template for a reverse transcription using the SuperScript III RT kit (Invitrogen) to synthesize cDNA. A qRT-PCR analysis of three replicates of the cDNA samples was performed using the SYBR Green Master Mix (Applied Biosystems, Foster City, CA, United States) and the CFX96 Real-Time System (Bio-Rad, Hercules, CA, United States).

## Results

### Variation in FER resistance among RILs

A total of 300 RILs were evaluated in terms of their FER resistance under field conditions in 2019, 2020, and 2021 in Changping, Shunyi, Xinxiang. The phenotypic variations are listed in Table 1. Transgressive segregation of FER resistance was observed in the RILs, and a wide range of variations was detected among families. The FER resistance among lines, which ranged from completely susceptible to highly resistant, was almost normally distributed, suggesting FER resistance is a quantitatively inherited trait. An analysis of the contributions of environmental and genetic factors to FER resistance revealed *H^2^* was as high as 95.46%, implying that the phenotypic variance in the RIL population was predominantly controlled by genetic factors (Table 2).

**Table 1:** Phenotype of parental lines and a recombinant inbred lines (RIL) population based on eight replicates.

Sites	Qi319	Ye478	RILs					
			Mean ± SD	Diseased area	Skewness	Kurtosis	CV%	*H^2^*%
E1	0.27	0.37	0.25613 ± 0.006424	0.031–0.687	1.04	1.52	2.51	95.46
E2	0.22	0.54	0.39357 ± 0.008269	0.098–0.877	0.63	0.35	2.1	
E3	0.21	0.68	0.24527 ± 0.007687	0.035–0.701	0.83	0.44	3.13	
E4	0.29	0.72	0.24517 ± 0.007687	0.01–0.687	1.8	5.02	3.52	
E5	0.11	0.45	0.16113 ± 0.005668	0.118–0.9	0.15	0.57	1.88	
E6	0.2	0.6	0.26781 ± 0.006705	0.027–0.647	0.87	0.5	2.5	
E7	0.16	0.38	0.28497 ± 0.006172	0.082–0.774	0.93	1.41	2.17	
E8	0.18	0.39	0.26235 ± 0.006131	0.035–0.643	0.8	0.88	2.34	

SD = standard deviation, CV = coefficient of variation, and H^2^ = generalized heritability.

**Table 2:** Analysis of variance of Fusarium ear rot resistance.

Source	df	SS	MS	*F* value	*Pr* > *F*	*H*^2^ (%)
Genotype (G)	364	42.92806984	0.117934258	7.796953017	2.4687E-229	95.46%
Environment (E)	7	16.32332348	2.331903355	154.168443	3.4342E-190	
G × E	2,548	700.7287705	0.275011291	1202.044106	0	
Residuals error	2,919	38.5402462	0.015125685			

df = degree of freedom, SS = sum of squares, MS = mean square deviation, and H^2^ = generalized heritability.

### Mapping of QTL for FER resistance in the RIL population

Seventeen QTL were identified in eight environments (Table 3). The QTL, which were located on six chromosomes, explained 3.88–15.62% of the phenotypic variation, tentatively designated as *qFER1.03*, *qFER1.05*, *qFER1.06*, *qFER1.07*, *qFER3.06*, *qFER4.07*, *qFER5.05*, *qFER7.02*, *qFER8.05*. Among the FER resistance QTL, *qFER1.03* had the strongest effect and the highest LOD value, which was detected in Shunyi, Changping, Xinxiang in 2019, Changping in 2020 and Shunyi in 2021, could explain 4.99% to 15.4 of the phenotypic variation. *qFER4.07* was detected in Xinxiang in 2019 and Shunyi in 2021, explaining 4.5% of the phenotypic variation. *qFER1.05* was detected in Xinxiang in 2020 and Shunyi in 2021, explaining 9.2% of the phenotypic variation. *qFER1.06*, detected in Shunyi in 2019 and Xinxiang in 2020, explained 4.6 to 5.8% of the phenotypic variation. Moreover, using a LOD threshold of 3.0 and a 95% confidence interval, two QTL, on average, were detected on chromosome 1. *qFER1.03* had LOD value of 6.49 and accounted for as high as 15.62% of the phenotypic variation. Based on the B73 RefGen_v3 sequence, *qFER1.03* was localized in the 41–49 Mb intervals.

**Table 3:** Quantitative trait loci (QTL) mapping for Fusarium ear rot resistance using a recombinant inbred line population derived from Qi319 and Ye478.

E	Chromosome	^a^Peak position	^b^Interval (B73_V3, Mb)	LOD	^c^PVE (%)	Add
E1	1	43.5	43–44	6.1735	10.8112	0.0536
E2	1	43.5	43–44	9.8834	15.4031	0.0467
	3	187.6	187.1–188.1	3.5259	5.2438	0.0264
E3	1	43.5	43–44	4.3553	4.9804	0.0279
	1	187.5	187–188	4.0032	4.5815	0.0261
	4	169.35	168.85–169.85	3.9803	4.5449	0.0262
	8	161.95	161.45–162.45	3.6001	4.1217	0.0245
	1	208.5	208–209	6.0969	8.3916	0.035
E4	1	41.5	41–42	4.0072	5.9042	0.0281
	1	129.5	125–138	6.0185	9.1556	0.0339
E5	1	85.5	85–86	6.118	9.2149	0.032
	1	193.5	192–194	3.8964	5.7595	0.0256
E6	1	43.5	43–44	9.8594	10.9206	0.0505
	4	168.35	166.85–168.85	4.3667	4.6294	0.0322
	5	178.45	177.95–178.95	3.6582	3.8835	0.0291
E7	1	48.5	48–49	9.2732	13.571	0.05
E8	7	108.35	107.85–108.85	4.4657	6.1587	−0.0368
BLUP	1	40.5	40–41	6.491	15.625	0.0262
	1	187.5	187–188	4.889	11.571	0.0218

^a^Peak position = position with the highest logarithm of odds (LOD) value.

^b^Interval = interval between two markers in the B73 RefGen_v3 genome sequence.

^c^PVE = phenotypic variance explained by a single QTL; ADD = additive effects of a QTL.

### Validation and mapping of the *qFER1.03* locus using a CSSL population

**Figure 1:**
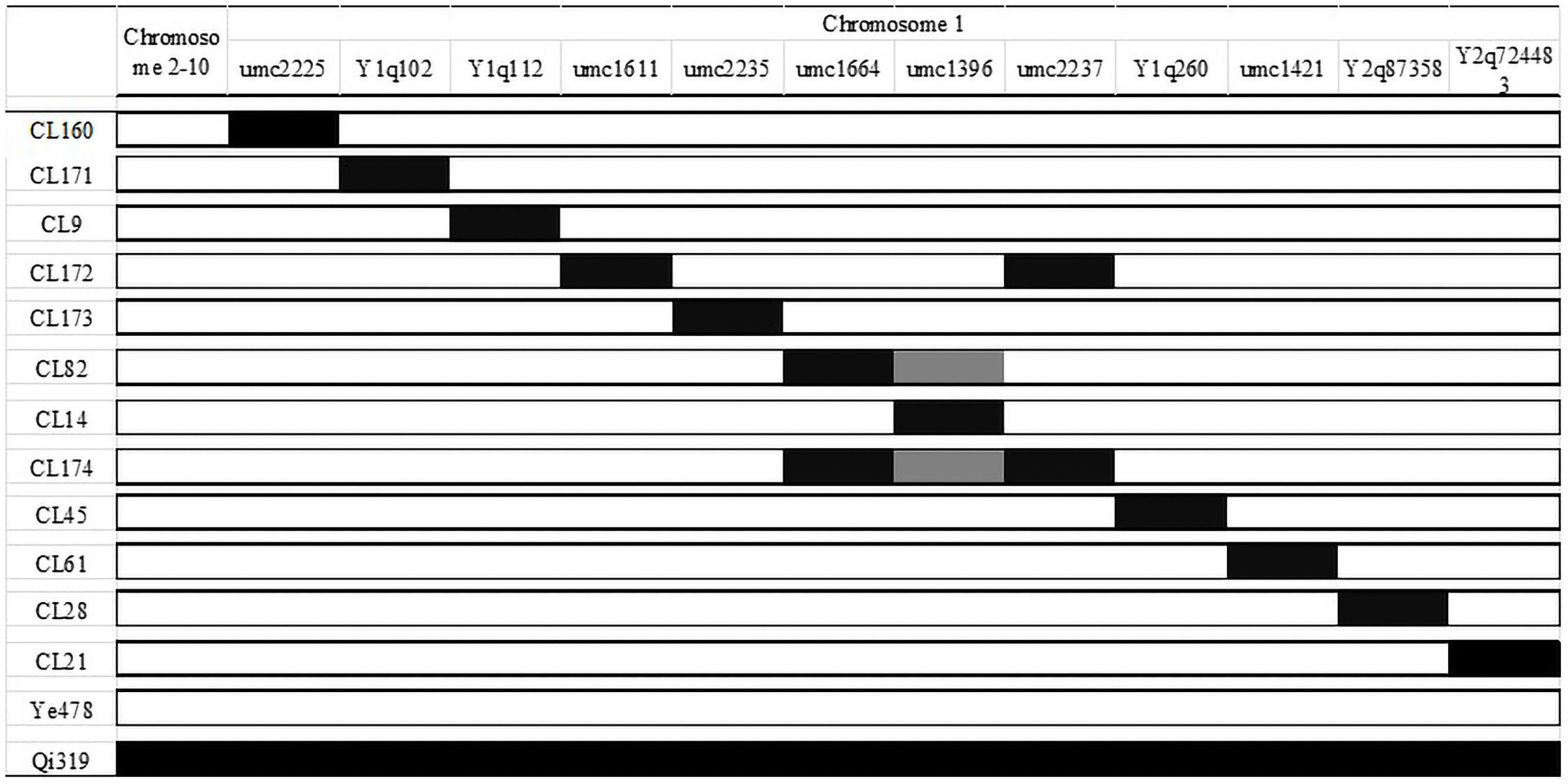
Identification of FER resistance QTL in the CSSL population.

The effect of *qFER1.03* was investigated using a CSSL population (derived from a cross between Qi319 and Ye478) that covered the whole chromosome 1 segments. The 12 selected CSSLs were obtained using the MAS method (BC_5_F_3_) by backcrossing for five generations and self-crossing for three generations. These 12 CSSLs had average background recovery rates of 97–99%. The QTL mapping results for the RIL population and 12 CSSLs covering chromosome 1 were used to map *qFER1.03*. The genotypes of the 12 CSSLs are presented in Figure 1, whereas the phenotypic data are provided in Table 4.

**Table 4:** Comparison of the FER resistance between the CSSLs and the susceptible control line Ye478.

CSSL	Background recovery	Diseased area	Resistance Growth (%)	Bin	Marker
CL160	0.98	0.63	0.07	1.02	bnlg1614-umc2225
CL171		0.35	0.45	1.03	Y1q25-bumc1144
CL9	0.99	0.71	−0.04	1.04	Y1q102-Y1q112
CL172		0.81	−0.19	1.05	umc1076-umc1611
CL173		0.66	0.03	1.05, 1.06	umc1611-umc1590
CL82	0.98	0.88	−0.29	1.06	umc2235
CL14	0.99	0.94	−0.38	1.06	umc1664-umc1709
CL174		0.67	0.01	1.06, 1.07	umc1254-umc2237
CL45	0.97	0.88	−0.29	1.07, 1.08	umc2505-umc2240
CL61	0.98	0.69	−0.01	1.09	Y1q260-Y1q272
CL28	0.98	0.69	−0.01	1.11	umc1421-bnlg2123
CL21	0.98	0.89	−0.31	1.11	Y2q87358-Y2q724483
Ye478		0.68			

**Figure 2:**
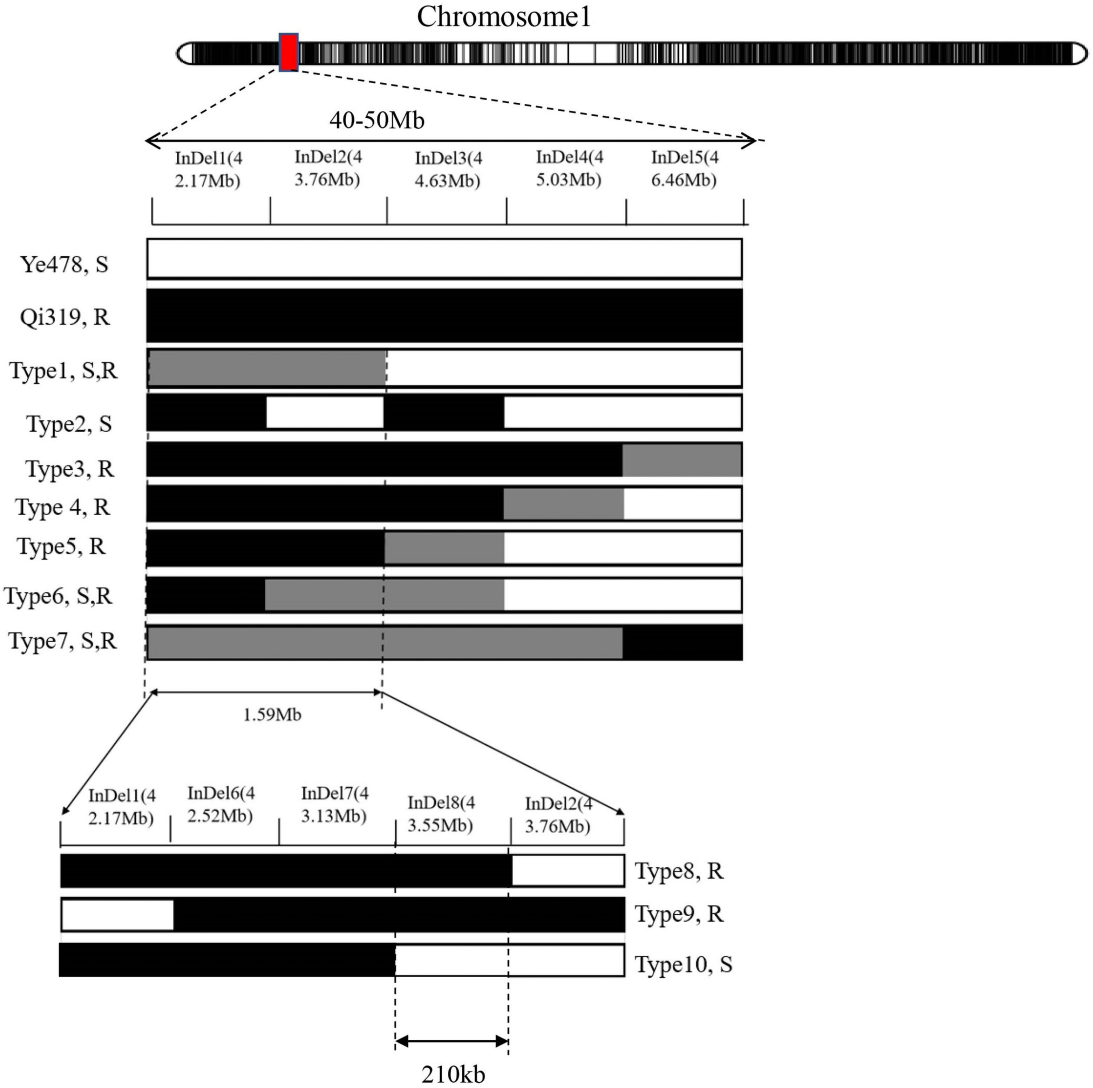
**(A)** Narrowing of the *qFER1.03* interval using an F_2_ hybrid generation derived from a cross between CL171 and Ye478. Black and gray rectangles represent homozygous Qi319 alleles and homozygous Ye478 alleles, respectively. Light gray indicates heterozygous alleles of Qi319 and Ye478. S and R refer to susceptible and resistant, respectively. **(B)** Narrowing of the *qFER1.03* interval using an F_3_ inbred generation derived from the selfing of Type 1.

Line CL171 was significantly more resistant to FER than the susceptible parent Ye478. The localization of the resistance locus was consistent with the interval revealed by the analysis of the RIL population, confirming that *qFER1.03* contributes to maize FER resistance. The fragment carrying *qFER1.03* was narrowed to 40–50 Mb on chromosome 1, flanked by markers between mk194 and mk210. On the basis of the whole-genome resequencing data for the two parents, primer pairs were designed for 243 InDel markers between mk194 and mk210, of which five (InDel 1–InDel 5) specific for one of the two parents were selected to detect the target interval. The F_2_ progeny obtained from the self-crossing of Ye 478 and CL171 were used for fine mapping. Additionally, 53 genotypes were identified. Finally, seven individual plants (Type 1–Type 7) were selected for a phenotypic analysis. The target fragment was located between 42.17 Mb and 43.76 Mb of chromosome 1 and was flanked by markers InDel 1 and InDel 2 (Figure 2). The F_3_ families derived from the selfing of Type 1 were used to further narrow the target interval. Three polymorphic InDel markers (InDel 6–InDel 8) were detected between 42.17 Mb and 43.76 Mb. Three individual plants were selected for a phenotypic analysis (Type 8–Type 10). Finally, *qFER1.03* was located between markers InDel 8 and InDel 2, with physical distances of 43.55 and 43.76 Mb, respectively (Figure 2).

### Integration of DEG and QTL data

Seven annotated genes were detected in the target interval between 43.55 Mb and 43.76 Mb on chromosome 1 following a search of MaizeGDB. According to the RNA-seq analysis, only one gene (*GRMZM2G017792*) was differentially expressed between Ye478 and Qi319. In MaizeGDB, *GRMZM2G017792* is named *mpk3* (MAP kinase 3). Additionally, its transcription and the activity of the encoded protein are upregulated by ABA and H_2_O_2_. Therefore, *GRMZM2G017792* was identified as a potential candidate gene for FER resistance. The STRING online program and RNA-seq data revealed an interaction between the proteins encoded by *GRMZM2G017792* and *GRMZM2G164405*, which is annotated in MaizeGDB as a 1-aminocyclopropane-1-carboxylic acid synthase 2 gene that is involved in limiting ethylene biosynthesis. Therefore, *GRMZM2G017792* may be related to ethylene synthesis.

### *GRMZM2G017792* was confirmed as a FER resistance-related gene by VIGS

**Figure 3:**
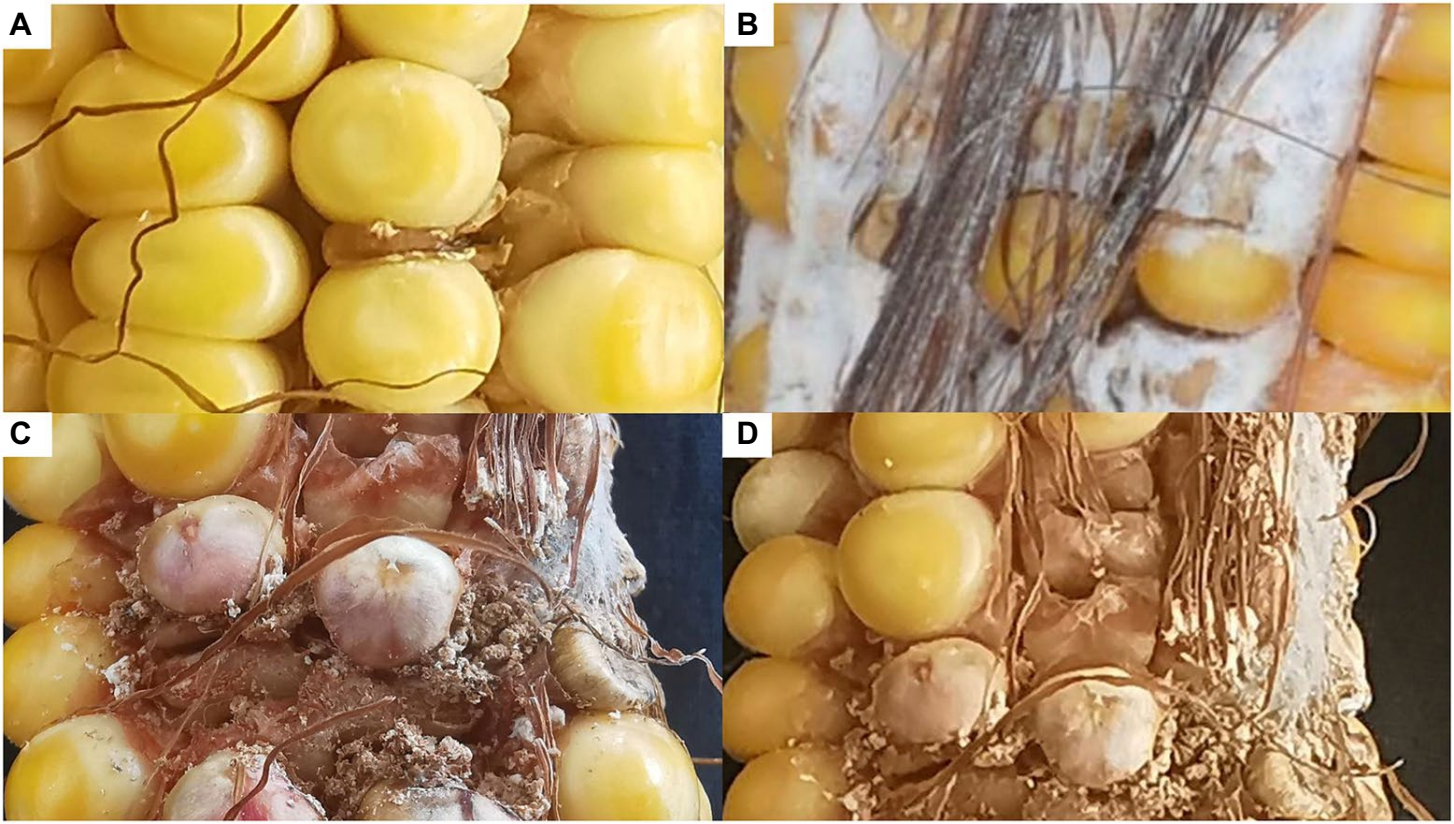
Incidence and severity of FER among CL171 and the gene-silenced materials. **(A)** CL171; **(B)** CL171-1; **(C)** CL171-2; **(D)** CL171-3.

**Figure 4:**
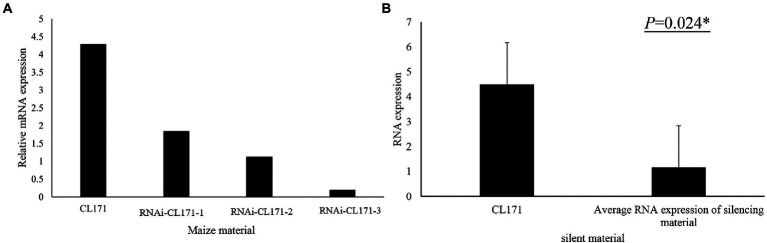
**(A)** Expression of *GRMZM2G017792* in CL171 and gene-silenced materials. **(B)** Significant difference (*p* = 0.024) in the RNA expression between CL171 and the gene-silenced materials (average).

To confirm its contribution to FER resistance, *GRMZM2G017792* was silenced in maize plants by VIGS. An analysis of FER incidence and severity in Sanya (Hainan) at 3 week post-inoculation indicated that all examined corn kernels were infected with *F. verticillioides*, with mycelia covering the cobs of CL171-1, CL171-2, and CL171-3, which differed regarding the extent of gene silencing. The grains were shriveled at the inoculation site in line CL171, but mycelial growth was restricted (Figure 3). Furthermore, CL171 was significantly more resistant to FER than CL171-1, CL171-2, and CL171-3, in which *GRMZM2G017792* was silenced. More specifically, the average RNA expression was significantly smaller (*p* = 0.024) for CL171 than for the gene-silenced materials (Figure 4).

## Discussion

Nowadays, Fusarium ear rot has been one of the most important diseases on maize in China. The breeding for FER resistance is pressing and has been an indispensable target trait. The mining of stable FER resistance genes/loci will facilitate resistance breeding. The accuracy of phenotypic value has a great influence on gene mapping. The performance of precise identification of maize resistance to ear rot was proven to be a difficult issue because of the genetic complexity and sensitive dependence of FER on environmental conditions. Therefore, all plants were artificially inoculated with pathogen spore suspension 5–7 days after silking to ensure that pathogen infection occurred at almost the same stage of kernel development among all individuals, and the Automatic Imaging System for Ear Rot was used to analyze the diseased area of inoculated ears. Compared with previous researches on the incidence and severity of maize ear rot on the basis of visual observations and estimations, the Automatic Imaging System for Ear Rot enabled a more accurate calculation of the diseased ear area. Using this method, we ensured that experimental uniformity and pathogen infection occurred at almost the same stage of kernel development among individuals.

Although some studies have reported FER-resistant loci derived from various inbred lines and mapped at different intervals (Zhang et al., 2007; Ding et al., 2008; Li et al., 2011). However, the newly identified genes/loci insensitivity to environments are needed for FER-resistant breeding. In this study, nine QTL associated with FER resistance overlapped regions in bins 1.03, 1.05 to 1.07, 3.06, 4.07, 5.05, 7.02 and 8.05. Among the intervals of bin 1.03, 3.06, 4.07, 7.02 overlapped with those reported in previous studies (Zhang et al., 2007; Chen et al., 2016; Wen et al., 2021), the major QTL at bin1.03 detected in five different environments explained the phenotypic variation as high as 15.4% Therefore, this QTL was selected for further fine mapping.

A combined analysis of RILs and CSSLs is a powerful approach for dissecting the QTL for FER resistance in maize. The CSSLs with the same genetic background can be used to mendelize QTL and substantially improve the localization of individual QTL. After preliminarily localizing QTL in a RIL population, we more precisely mapped QTL using CSSLs. This approach is useful for detecting FER resistance-related genes. The isolated QTL can be quickly localized by identifying QTL-linked markers in a CSSL population (Yung et al., 2012). We used SSR markers to select 12 CSSLs with different genetic backgrounds for chromosome 1, but identical backgrounds for the remaining chromosomes, to eliminate the effects of other FER resistance-related genes. These CSSLs were used for reconfirming and precisely localizing the newly identified major QTL *qFER1.03*. Line CL171 was resistant to FER, whereas the other CSSLs were susceptible. Therefore, CL171 may be used to construct a secondary backcross population for the fine mapping and cloning of the gene mediating FER resistance. Therefore, CL171 can be used to construct a secondary backcross population for fine positioning and cloning of the significant gene for FER resistance. This strategy was feasible for laying a foundation in FER resistance breeding.

Transcriptomic data provide researchers with important information for mining candidate genes for maize ear rot resistance. In the current study, only one gene (*GRMZM2G017792*) in the QTL region was differentially expressed between the parents susceptible (Ye478) and resistant (Qi319) to FER. The mitogen-activated protein kinase cascade is a signal transduction pathway that is common in eukaryotes. It plays an important role in plant responses to biotic and abiotic stresses. Recent studies on *Arabidopsis thaliana* confirmed that MPK3 and MPK6 are important for leaf stomatal development, petal shedding, and ovule development. Moreover, they are activated by various stimuli (e.g., salt stress and pathogen infection) during the regulation of diverse defense responses, making them crucial for plant disease resistance. Meng et al. (2013) reported that MPK3/MPK6 in *A. thaliana* phosphorylate ERF6 (ethylene-responsive factor), thereby activating defense-related gene expression and enhancing the resistance to *Botrytis cinerea*. Li et al. (2021) observed that MPK3/6 phosphorylates ERF72 to regulate the transcriptional activation of *PAD3*, *CYP71A12*, and *WRKY33*.

In a previous study involving CMV-ZMBJ-based VIGS in maize line B73, a vascular puncture inoculation method resulted in a viral infection rate of approximately 59%, but only about 60% of the infected plants exhibited different degrees of gene silencing. Moreover, the gene-silencing efficiency in five young systemic leaves varied from 25 to 78% among individual plants over 60 days (Wang et al., 2016). The relatively low viral infection rate associated with the vascular puncture inoculation method is not unique to CMV, with similar results obtained for other VIGS vectors (e.g., BSMV and MRFV; Jarugula et al., 2018; Mlotshwa et al., 2020). In contrast, the Pr-CMV-VIGS system can silence the target gene in maize for relatively long periods. More specifically, Pr CMV: *ZmIspH* was used for the highly efficient and durable silencing of *ZmIspH* (59.4–87.3%) for over 105 days, which is the longest period of VIGS in maize reported to date (Li et al., 2021).

In the present study, we silenced the candidate gene *GRMZM2G017792* using the Pr-CMV-VIGS system, which resulted in a distinct decrease in the FER resistance of CL171. Pyramiding several disease resistance-related genes in a single variety and developing multiline cultivars are the most effective methods for increasing the durability of maize FER resistance. In this study, *GRMZM2G017792* was identified as a candidate FER resistance-related gene, which may be useful for maize breeding. More specifically, it may be exploited to decrease the incidence and severity of FER outbreaks. Future studies will need to clone and functionally characterize the candidate gene. Furthermore, clarifying the molecular mechanism underlying FER resistance in maize will enhance the breeding of superior inbred lines and FER-resistant hybrids.

## Data availability statement

The data presented in the study are deposited in the NCBI repository, accession numbers are SAMN29837145, SAMN29837146, SAMN29837147, SAMN29837148, SAMN29837149, SAMN29837150, SAMN29837151, SAMN29837152, SAMN29837153, SAMN29837154, SAMN29837155, SAMN29837156, SAMN29837157, SAMN29837158, SAMN29837159, SAMN29837160, SAMN29837161, SAMN29837162, SAMN29837163, SAMN29837164, and SAMN29837165.

## Author contributions

YX, JW, and CD conceived and designed the experiments. XL discussed experimental scheme and revised the article. YX, BW, LZ, and ZZ performed RILs phenotype data collecting. YX, LZ, XL, and SS analyzed the RILs data and conducted the QTL identification and further fine mapping. YX, JW, XL, and LZ constructed the sequence library of the Ye478 and Qi319. YX, WW, and CD analyzed the data and discussed the article. YX and CD wrote the paper. All authors contributed to the article and approved the submitted version.

## Funding

This project was supported by the National Key Research and Development Program of China (2021YFD1200702 and 2016YFD0100103), the Project of Sanya Yazhou Bay Science and Technology City (SKJC-2020-02-001), and Agricultural Science and Technology Innovation Project of Chinese Academy of Agricultural Sciences (CAAS-ASTIP-2017-ICS).

## Conflict of interest

The authors declare that the research was conducted in the absence of any commercial or financial relationships that could be construed as a potential conflict of interest.

## Publisher’s note

All claims expressed in this article are solely those of the authors and do not necessarily represent those of their affiliated organizations, or those of the publisher, the editors and the reviewers. Any product that may be evaluated in this article, or claim that may be made by its manufacturer, is not guaranteed or endorsed by the publisher.
